# In This Issue

**DOI:** 10.1002/cfg.84

**Published:** 2001-06


**Comparative and Functional Genomics**
*Comp Funct Genom* 2001; **2**: 123.
**DOI:** 10.1002/cfg.84

### In this Issue

### A genomic approach to identifying and classifying yeast genes involved in cell wall formation and its regulation de Groot *et al*. have systematically screened 620 non-essential single-gene yeast deletants (generated by EUROFAN1) for cell wall-related phenotypes. They tested for altered sensitivity to Calcofluor white, SDS or sonication treatment and looked for abnormal morphology. 145 mutants showing at least one phenotype were selected; these mutants then underwent further screens to determine which genes were related to which stages of cell wall formation or its regulation.

### A reannotation of the *Saccharomyces cerevisiae* genome, using the same approach as for the complete sequence of the *Schizosaccharomyces pombe* genome

Woods *et al*. have applied the genome analysis pipeline derived for the annotation of the *S. pombe* genome to the *S. cerevisiae* genome, to define an up-to-date, non-redundant gene set with consistent annotations and accurate orphan numbers. This analysis refines the gene set, divides the orphans into 'hypothetical' and 'very hypothetical' proteins (based on a series of criteria that relate to their coding likelihood) and identifies several gene prediction errors.

### Combining genomics, metabolome analysis and biochemical modeling to understand metabolic networks

In his review, Oliver Fiehn discusses new ways towards understanding the functions of genes involved in metabolism. The crux of this approach is the profiling of metabolites, typically producing either accurate quantification of selected target metabolites, or fingerprints of general metabolic changes without determining the identities of individual metabolites. These approaches (and others) are discussed, along with data analysis issues relating to pattern recognition and network modeling.

### Definition of the gene content of the human genome: the need for deep experimental verification

Andrew Simpson and colleagues discuss the apparently low estimates of the human gene count arising from the two recently published drafts of the human genome. They describe the shortcomings of gene identification from genomic sequence data and the various techniques that have been used to estimate the human gene count, highlighting some evidence that there may be more human genes, which remain as yet undetected. They also discuss the need for further transcript sequencing to allow the compilation of a meaningful human gene catalogue.

### Interview with Duncan Campbell: After the draft sequence, what next for the Human Genome Mapping Project Resource Centre?

Dr R. Duncan Campbell is the Director of the Medical Research Council (MRC) funded UK Human Genome Mapping Project Resource Centre (HGMP-RC), on the Wellcome Trust Genome Campus at Hinxton, near Cambridge, UK. The Centre provides the research community with biological and bioinformatics resources, and has research groups supporting these activities and investigating separate projects. He talks to us about the work of the centre, and how it will be changing in the next few months, in response to the genome drafts and the swing towards functional genomics.

### Meeting Review: IBC Proteomics

We present a review of this recent proteomics meeting by Philip Masterson of KuDOS Pharmaceuticals. The sessions covered 2D-gel electrophoresis and alternative separation techniques, strategies for functional proteomics, developments in proteo-informatics, progress made with protein arrays, and reports of successes so far in applying these techniques.

Copyright © 2001 John Wiley & Sons, Ltd.

